# Combined Exercise and Ursolic Acid Improve Hippocampal Neuronal Markers and Exploratory-Locomotor Behavior in Aged Diabetic Rats

**DOI:** 10.1155/jare/9916781

**Published:** 2025-08-04

**Authors:** Safoura Alizade, Abbas Ali Gaeini, Mohammad Faramarzi

**Affiliations:** ^1^Department of Exercise Physiology, Faculty of Sport Sciences, University of Isfahan, Isfahan, Iran; ^2^Department of Exercise Physiology, Faculty of Sport Sciences, University of Tehran, Tehran, Iran

**Keywords:** exercise, neuronal biomarkers, open field test, type 2 diabetes mellitus‏, ursolic acid

## Abstract

**Background:** Diabetes mellitus is linked to progressive cognitive decline and motor impairments, especially among the aging population, highlighting the importance of early detection through reliable neuronal biomarkers. Proteins such as neurofilament light chain (NFL), neurogranin (Ng), and visinin-like protein 1 (VILIP-1) have emerged as indicators of neurodegeneration and associated behavioral changes. This study examined the effects of combined endurance and resistance exercise, along with ursolic acid (UA) supplementation, on hippocampal neuronal biomarkers and exploratory-locomotor behavior in aged diabetic rats.

**Methods:** In this experiment, 21-month-old male Wistar rats were assigned to seven groups. Diabetes was induced using a single intraperitoneal dose of streptozotocin (STZ) (30 mg/kg) in combination with a high-fat diet (55% fat, 31% carbohydrate, and 14% protein). Interventions included endurance training (60%–75% vVO_2max_), resistance training (60% MVCC), and daily oral UA administration (250 mg/kg) over eight weeks. Neuronal biomarkers (NFL, Ng, and VILIP-1) were measured in hippocampal tissue via western blot, and exploratory and locomotor behavior was assessed using the open-field test.

**Results:** The results showed that UA supplementation combined with resistance training significantly reduced the levels of neuronal biomarkers NFL (*p* < 0.001), Ng (*p* < 0.01), and VILIP-1 (*p* < 0.001) in diabetic rats compared to untreated diabetic controls.

**Conclusion:** The study demonstrated that diabetes leads to a marked elevation in NFL, Ng, and VILIP-1 protein levels, while a combined intervention of exercise and UA mitigated neurodegenerative changes and improved exploratory-locomotor outcomes.

## 1. Introduction

Diabetes mellitus is a widespread metabolic disorder that continues to rise globally, particularly among the aging population [1]. In older adults, diabetes often coexists with chronic conditions such as hypertension, coronary artery disease, and cognitive decline and behavioral alterations [2, 3]. Given the brain's high energy demands, its function depends heavily on consistent glucose delivery through the blood–brain barrier (BBB). Metabolic disruptions such as chronic hyperglycemia and insulin resistance impair this transport system, potentially leading to neuronal dysfunction and related behavioral impairments [4]. In addition, hyperglycemia has been associated with vascular ischemia [5, 6], accumulation of amyloid β, and impaired insulin signaling in the brain changes that may contribute to Alzheimer's-like neuropathology [7, 8]. Indeed, diabetes and Alzheimer's disease (AD) share multiple overlapping features, including alterations in insulin-like growth factor signaling and neuronal insulin resistance [9].

Recently, considerable attention has been drawn to molecular biomarkers that may reflect early signs of neurodegeneration and associated behavioral alterations [10]. Among these, neurofilament light chain (NfL), neurogranin (Ng), and visinin-like protein 1 (VILIP-1) have emerged as key indicators of neuronal integrity, axonal injury, and synaptic function [11]. NfL is a structural axonal protein released during neuronal damage [12]; Ng plays a regulatory role in synaptic plasticity via calmodulin signaling [13]; and VILIP-1, part of the neuronal calcium sensor family, reflects calcium-mediated neurotoxicity [14]. Several studies report elevated levels of these biomarkers in cognitive impairment, suggesting that they could serve as useful early indicators in populations at risk, such as those with type 2 diabetes [14–17]. A study by Frederick et al. reported that only 19% of the variation in serum NfL levels can be attributed to factors such as age, declining kidney function, and glycemic control assessed through hemoglobin HbA1C [18]. However, there is still limited research examining the relationship between biomarkers of neuronal and synaptic integrity and cognitive deterioration in diabetes.

Dietary changes and physical exercise are fundamental interventions aimed at slowing the progression of metabolic disturbances associated with prediabetes, while also serving as adjuncts to pharmacological treatments for type 2 diabetes mellitus (T2DM) [19, 20]. Physical exercise remains a cornerstone of diabetes management due to its positive effects on glucose regulation, insulin sensitivity, and cardiovascular health [21]. Emerging findings suggest that exercise may also offer neuroprotective benefits, particularly among the elderly [3, 22]. Endurance and resistance training have both been associated with increased neurogenesis and improved cognitive outcomes in various animal models, although human results remain inconsistent [23, 24]. One potential reason for the variability is the heterogeneity of exercise protocols and study populations. Furthermore, the effects of exercise on specific neuronal biomarkers such as NfL, Ng, and VILIP-1 have not been conclusively established, with some studies reporting improvement and others observing no significant change [25–27]. These findings highlight significant variability in how individuals respond to exercise, emphasizing the need for more detailed studies to understand the factors that may influence the effects of physical activity on these outcomes.

Ursolic acid (UA), a naturally occurring pentacyclic triterpenoid found in herbs and fruits like apple peel and rosemary, has garnered attention for its broad pharmacological effects including antioxidant, anti-inflammatory, and antidiabetic properties [28]. Experimental studies suggest that UA may protect against neurodegeneration by enhancing antioxidant enzyme expression, suppressing inflammatory pathways, and regulating insulin signaling in the brain [29, 30]. For instance, UA has been shown to activate PI3K/Akt signaling and inhibit FoxO1 in hippocampal neurons [31]. Despite this promising profile, no study has yet explored the effect of UA supplementation on NfL, Ng, or VILIP-1 expression in a diabetic model. Additionally, it remains uncertain whether exercise combined with UA supplementation can mitigate diabetes-related neuronal and behavioral dysfunction by influencing biomarkers of neuronal injury.

Therefore, the aim of the present study was to examine the effects of endurance and resistance training, with or without UA supplementation, on hippocampal levels of NfL, Ng, and VILIP-1 in aged diabetic rats. The study also assessed whether these interventions could lead to improved exploratory-locomotor behavior and neuronal marker profiles, thereby shedding light on their potential neuroprotective mechanisms in the context of diabetes and aging.

## 2. Methods

### 2.1. Ethics

The study was authorized by the Ethics Committee for Laboratory Animals at Shahrekord University (IR.SKU.REC.1399.001, ethics.research.ac.ir). All experimental procedures conformed to applicable guidelines and regulations, including the ARRIVE guidelines for reporting in vivo experiments established by Percie du Sert et al. in 2020, ensuring ethical treatment and use of research animals [32].

### 2.2. Study Sample

#### 2.2.1. Facilities and Participants

Thirty-five male Wistar rats, averaging 21 months in age and weighing 427 ± 44 g, were sourced from the Pasteur Institute in Tehran, Iran. Upon their arrival in the laboratory, the rats were kept in a controlled setting featuring a 12-h light–dark cycle (light from 08:00 to 20:00), with the temperature regulated at 20 ± 2°C and humidity maintained at 50 ± 5%. They underwent a 1 week acclimatization period, followed by an additional week to get accustomed to treadmill activity (10–15 min daily at speeds of 5–10 m/s).

**Figure 1:**
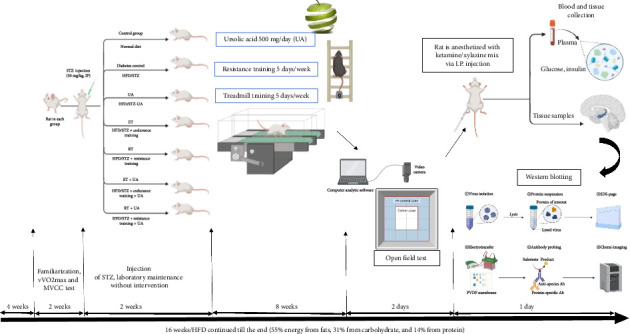
Experimental timeline: Before starting the study, all the rats except the control group were fed for 4 weeks with an HFD. After familiarizing the animals with the laboratory environment and treadmill, diabetes was induced by STZ injection (30 mg/kg, IP) in diabetic groups. Then, all rats were kept in the laboratory for 2 weeks (without any intervention). Treatment with endurance/resistance training and UA began for 8 weeks. Thereafter, in the final week, the open field test was used to assess learning and memory and then they were sacrificed for molecular studies.

The rats were then randomly allocated into seven groups: sedentary old nondiabetic (C), sedentary HFD/streptozotocin (STZ)-induced T2DM (DM), sedentary HFD/STZ-induced T2DM plus UA (UA), endurance-trained HFD/STZ-induced T2DM (ET), resistance-trained HFD/STZ-induced T2DM (RT), endurance-trained HFD/STZ-induced T2DM plus UA (ET + UA), and resistance-trained HFD/STZ-induced T2DM plus UA (RT + UA). A schematic depicting the study's implementation process over 16 weeks is provided in Figure 1.

### 2.3. Induction of Diabetes

After 4 weeks of feeding, all rats except those in the control group were given a high-fat diet consisting of 55% fat, 31% carbohydrates, and 14% protein, totaling 5.2 kcal/g. In contrast, the control group received a standard basal diet with 10% fat, 75% carbohydrates, and 15% protein, sourced from Royan Company in Isfahan, Iran. Following this dietary phase, the experimental group animals were administered an intraperitoneal injection of STZ (Sigma-Aldrich, St. Louis, MO, USA) at a dose of 30 mg/kg, dissolved in 0.1 M sodium-citrate buffer at pH 4.4 [33].

Seventy-two hours after the induction of diabetes, blood samples were collected from the tails of the remaining rats and analyzed using a glucometer (Bionime, GM110, Switzerland). Rats with fasting blood glucose (FBG) levels of 200 mg/dL or higher were classified as diabetic. Following the STZ injection, a 2 week acclimatization period was provided for the rats in the laboratory without any interventions.

FBG levels were monitored at the first, second, fourth, sixth, and eighth weeks, while body weight (BW) was recorded weekly for all animals in each group throughout the study using a digital scale with an accuracy of 0.001 kg (Seca, Hamburg, Germany).

### 2.4. Supplementation

UA was sourced from a proprietary herbal supplier. The supplement was provided by a company specializing in healthy aging products (Knowledge-Based Company, Tehran, Iran). All animals in the UA, ET + UA, and RT + UA groups received daily treatment for 8 weeks alongside a high-fat diet that included 500 mg/mL of UA (0.5% w/v concentration, corresponding to 500 mg of UA extract per 30 mL of distilled water) [34].

### 2.5. Endurance Exercise Protocol

Two weeks after inducing diabetes, an endurance training program was initiated, taking place 5 days a week for 8 weeks at a moderate intensity. Each training session consisted of three parts: a warm-up (5 min at 40%–50% of velocity at maximal oxygen uptake [vVO_2max_]), a main exercise segment featuring moderate-intensity training (60%–75% of vVO_2max_), and low-intensity training (30%–40% of vVO_2max_), followed by a cool-down (5 min at 40%–50% of vVO_2max_).

The intensity and duration of the moderate-intensity training gradually increased from the first to the eighth week. In the initial week, the program included 4 repetitions at 60% vVO2max and 3 repetitions at 40% vVO_2max_. By the second week, this progressed to 4 repetitions at 65% vVO_2max_ and 3 repetitions at 40% vVO_2max_. In the third week, it consisted of 6 repetitions at 70% vVO_2max_ and 5 repetitions at 40% vVO_2max_. From the fourth to the eighth week, the routine escalated to 8 repetitions at 75% vVO_2max_ and 7 repetitions at 30% vVO_2max_, with each repetition lasting 2 min.

vVO_2max_ was measured to evaluate the exercise capacity of the diabetic rats. vVO_2max_ was determined using the standard incremental test outlined by Leandro et al. This test comprised 10 stages, each lasting 3 minutes with a zero incline. The treadmill speed began at 0.3 km/h in the first stage and increased by 0.3 km/h in each subsequent stage until the maximum running speed was reached, at which point the rats could no longer continue. Assessments of vVO_2max_ were performed in the fourth and eighth weeks of the study [35].

### 2.6. Resistance Exercise Protocol

The rats in the RT and RT + UA groups participated in designated exercises 5 days a week for 8 weeks, with the intensity set at 60% of their maximum voluntary carrying capacity (MVCC) after 5 days of acclimatization. Their exercise regimen involved climbing a 110 cm ladder featuring a 2 cm grid at an 85° incline. During the familiarization phase, the rats ascended the ladder without any additional weights. In the training phase, however, progressive resistance was introduced by attaching weights to their tails. The rats climbed the ladder repeatedly, completing either 14 or 20 ascents per session until they showed signs of fatigue. After reaching the top, they were given a 1 min rest period.

To establish the MVCC, a weight equivalent to 75% of their BW was attached to their tails, and the rats began climbing the ladder with this load. For each successful ascent, an additional 30 g was added until the rats reached total exhaustion, indicated by their inability to climb the ladder for three consecutive attempts. MVCC assessments were conducted at the beginning of the training and again after the fourth and eighth weeks [36].

### 2.7. Experiments of Behavioral Test

The open field test (OFT) was performed to assess exploratory behavior, locomotor activity, and anxiety-like responses. The adaptation phase involved introducing each animal to a novel environment free from any negative or positive stimuli. The animals were then permitted to explore the area for a specified duration, typically 5 minutes. A square wooden box measuring 50 × 60 × 40 cm served as the testing environment, with the floor marked by white lines that divided it into 16 equal squares of 4 × 4 cm.

Each rat was initially placed gently in the center of the field and allowed 5 min for unimpeded exploration. After each trial, the floor and walls were thoroughly cleaned to remove any residual odors that could influence subsequent trials. During the test, several parameters were recorded for each rat, including the time spent in the central area, the frequency of instances where the animal stood and stretched on its hind legs both with and without support from its forelimbs, and the total number of squares crossed during the five-minute exploration period [37, 38].

### 2.8. Tissue Samples

At the conclusion of the experiment, the rats were deeply anesthetized with a combination of ketamine (90 mg/kg) and xylazine (10 mg/kg). Blood samples were then collected and centrifuged to separate the serum, which was subsequently stored at −80°C for later analysis. In parallel, hippocampal tissue samples were rapidly excised, immediately frozen in liquid nitrogen, and preserved at −80°C until needed for further examination [39–41].

### 2.9. Western Blot Analysis

Western blot analysis was performed in accordance with standard protocols, utilizing polyclonal antibodies specific to VILIP-1 (sc-517633), Ng (sc-514922), NF-L (sc-20012), and β-actin (sc-47778). To accurately compare proteins of similar molecular weights, antibodies from different species were used, or the blot was stripped and reprobed as needed.

The intensity of the bands on the immunoblots was then quantified using densitometry with Image Studio Lite software, allowing for accurate assessment and comparison of protein expression levels.

### 2.10. Data Analysis

Data analysis was performed using SPSS 25 for Windows (SPSS Inc., Chicago, IL), with results presented as mean ± SEM. Prior to analysis, data were tested for normality and homogeneity using the Kolmogorov–Smirnov and Levene's tests, respectively.

Repeated measures were applied to variables such as blood glucose, weight changes, MVCC, and vVO_2max_. Additionally, a one-way analysis of variance (ANOVA) was conducted to compare the levels of research variables in the hippocampal histological analysis across different groups. Tukey's post hoc test was used to assess group differences further. A significance level of *p* < 0.05 was adopted, ensuring a rigorous and reliable interpretation of the experimental outcomes.

## 3. Results

### 3.1. Phenotype of Aging Diabetic Rats

**Figure 2:**
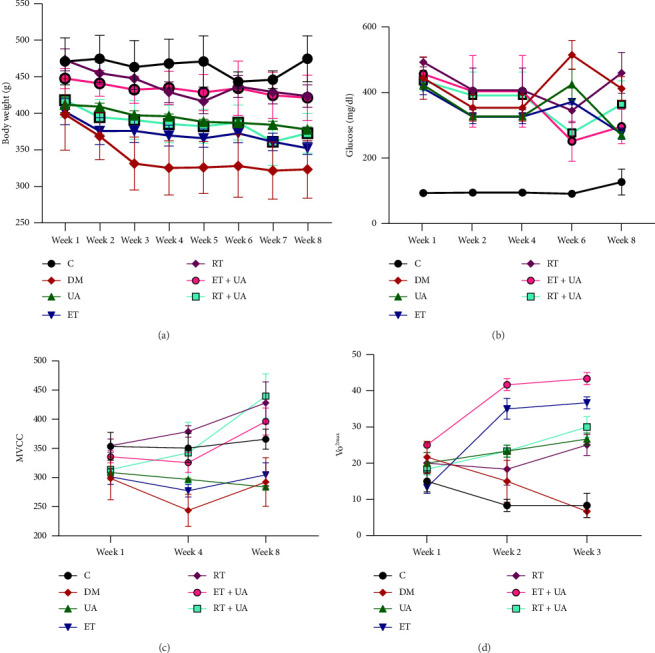
Phenotype of aging diabetic rats. (a) Body weight changes, (b) glucose concentration, (c) determining maximum voluntary carrying capacity (MVCC), and (d) running speed test for determining velocity at VO_2max_ (vVO_2max_). Values are expressed as mean ± SEM. Data were analyzed by repeated measures, *n* = 5 per group.

BW changes over the 8 week intervention period showed a significant time effect (*p*=0.001). Within-group analysis revealed a significant increase in BW in the RT + UA (*p*=0.035), RT (*p*=0.021), DM (*p*=0.042), and UA (*p*=0.046) groups by the eighth week compared to the first week (Figure 2(a)).

Additionally, glucose concentration significantly decreased in the ET + UA, ET, RT, UA, DM, and RT + UA groups compared to the control group (*p* ≤ 0.001) (Figure 2(b)). MVCC was notably higher in the RT group compared to the DM group (*p*=0.028). Within-group analysis also indicated a significant increase in MVCC in the RT + UA (*p*=0.031) and UA (*p*=0.046) groups at the posttest stage compared to pretest levels (Figure 2(c)). Furthermore, our results showed a significant improvement in vVO_2max_ with exercise training (both endurance and resistance) combined with UA consumption, compared to the control and DM groups (*p* < 0.05) (Figure 2(d)).

No significant differences in blood glucose concentration between groups were found throughout the study (*F* = 2.126, *p*=0.115) (Table 1). However, insulin levels showed a significant difference (*F* = 4.360, *p*=0.011). Rats treated with resistance training or UA supplementation exhibited a significant reduction in insulin expression (*p* < 0.01).

### 3.2. Resistance Training and UA Supplementation Enhance Neuronal Biomarkers in HFD/STZ-Induced Diabetic Rats

**Figure 3:**
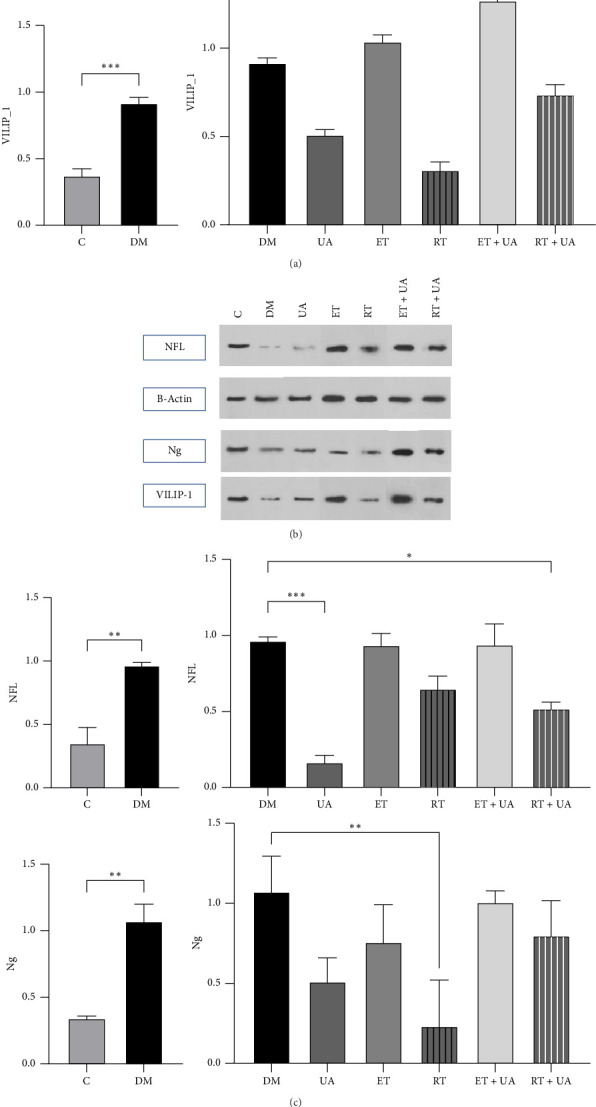
Western blot analysis of neuronal biomarkers in hippocampal tissue. (a) Expression levels of VILIP-1, (b) neurofilament light chain (NFL), and (c) neurogranin (Ng) proteins in the hippocampus of control, diabetic (DM), ursolic acid (UA), endurance training (ET), resistance training (RT), combined ET + UA, and combined RT + UA treated rats. Protein expression was assessed by western blotting and normalized to β-actin as a loading control. Data are presented as mean ± SEM (*n* = 5 per group). Statistical analysis was performed using one-way ANOVA followed by Tukey's post hoc test. ^∗^*p* < 0.05, ^∗∗^*p* < 0.01, ^∗∗∗^*p* < 0.001, ^∗∗∗∗^*p* ≤ 0.0001 vs. DM group; ^∗∗∗^*p* < 0.001, ^∗∗^*p* < 0.01 vs. control group.

Figure 3 presents the results of the western blot analysis for the neuronal biomarkers VILIP-1 (a), NFL (b), and Ng (c). A one-way ANOVA revealed a significant difference in hippocampal VILIP-1 protein expression among the experimental groups (*F* = 46.35, *p* ≤ 0.0001). Post hoc Tukey's test indicated that VILIP-1 levels were significantly decreased in the RT group and the UA group compared to the DM group (*p* < 0.0001 for both). Similarly, NFL protein expression varied significantly among groups (*F* = 16.90, *p* ≤ 0.001). Combined RT and UA treatment resulted in a significant reduction in NFL levels compared to the DM group (*p* < 0.05), while UA supplementation alone also significantly reduced NFL expression (*p* < 0.001). Regarding Ng levels, ANOVA showed significant differences (*F* = 6.782, *p*=0.003). Ng expression was significantly elevated in the DM group compared to the control group (*p* ≤ 0.01), while resistance training significantly decreased Ng levels relative to the DM group (*p*=0.03).

#### 3.2.1. OFT

**Figure 4:**
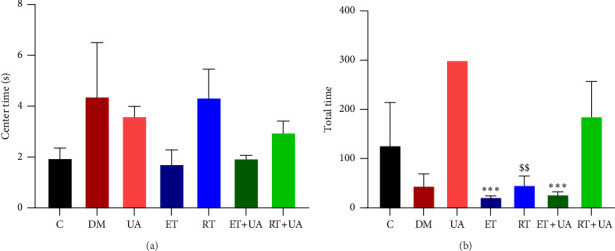
Open field test (a, b). Values are expressed as mean ± SEM. Data were analyzed by one-way ANOVA, *n* = 5 per group. ^∗∗∗^*p* ≤ 0.001 compared the ET and ET + UA to DU between groups. ^$$^*p* ≤ 0.015 compared the RT to UA between groups.

Diabetes is associated with behavioral alterations, including reduced exploration. We assessed exploratory-locomotor behavior in STZ-induced diabetic rats using the OFT. As shown in Figure 4, there were significant differences in exploratory activity among the groups based on total distance traveled (*F* = 5.562, *p*=0.004). STZ-induced diabetic rats in the ET + UA (*p*=0.009), ET (*p*=0.008), and RT (*p*=0.015) groups demonstrated significantly reduced exploratory activity compared to rats in the UA group (Figure 4(b)). However, as shown in Figure 4(a), no significant differences were found between groups in the time spent in the center area of the field (*F* = 1.393, *p*=0.284).

## 4. Discussion

In this research, we explored for the first time the impact of endurance/resistance training combined with UA supplementation on molecular markers related to neurodegeneration in the hippocampal tissue of diabetic rats, which were induced using STZ and a high-fat diet. Our findings indicated that both resistance training and UA supplementation had a statistically significant effect on insulin levels and insulin resistance. Heden et al.'s study also observed a marked reduction in insulin levels in the resistance training group [42, 43]. Moreover, in our study, administering 250 mg/kg of UA for 8 weeks led to a notable increase in BW and a significant reduction in plasma insulin levels when compared to the diabetic group. These outcomes align with previous studies by González-Garibay et al. and Tang et al., which showed that UA reduced blood glucose, insulin resistance, and hyperinsulinemia in the UA-treated group relative to the diabetic model group [44, 45].

The results demonstrated a significant increase in MVCC in both the RT and RT + UA groups after 8 weeks, compared to the other groups. Previous studies have shown that resistance training performed for 6–12 weeks, three times per week, with a load of 70%–80% of the one-repetition maximum, improves quadriceps muscle size and strength in individuals with type 2 diabetes [46]. Similarly, a study by Ebert and colleagues found that 12 weeks of UA supplementation resulted in dose-dependent improvements in both muscle strength and mass, which are consistent with the findings of the present study [47].

Additionally, the study revealed that after 8 weeks, vVO_2max_ increased in both the ET and ET + UA groups compared to the control group. Endurance training has been shown to induce mitochondrial adaptations, improve cardiovascular function, and enhance aerobic capacity, thereby optimizing skeletal muscle performance, especially in older adults [48]. Supporting these results, Bakhtiari et al. identified a relationship between mitochondrial count, myoglobin expression, red muscle mass, and aerobic respiration rate, suggesting that UA may contribute to increasing vVO_2max_ values [49].

UA's neuroprotective effects have also been demonstrated in cases of cerebral ischemia in mice [50]. In this study, we utilized the OFT to assess exploratory and locomotor behavior. Our results showed that administering UA (250 mg/kg) in combination with endurance training significantly reduced the total distance traveled in the OFT, suggesting that UA may modulate locomotor activity and improve learning abilities in rats. These findings support the protective effects of UA (250 mg/kg over eight weeks) against behavioral alterations in diabetic rats, as observed in the OFT. This aligns with a recent study on a T2DM rat model, where UA administration was found to prevent cognitive impairments and improve functional motor in the hippocampus [38, 51].

In diabetes, calcium homeostasis is often disrupted, with hyperglycemia being a key factor influencing calcium imbalances in critical organs such as skeletal muscles, the liver, heart, and brain, all of which respond to insulin [52]. These disruptions involve pathways reliant on the activation of calmodulin-dependent calcium kinase (calcium/calmodulin-dependent protein kinase II [CaMKII]), which is regulated by mitochondrial reactive oxygen species, calcium release from the endoplasmic reticulum, or hyperglycemia-induced oxygen-dependent glycosylation. Nevertheless, the precise molecular mechanisms linking hippocampal calcium dysfunction and cognitive deficits associated with hyperglycemia remain uncertain [53].

The results of our study indicate that diabetes caused a marked increase in the levels of NFL, Ng, and VILIP-1 within the hippocampal tissue of rats. These proteins, which are indicators of neural damage, including NFL, Ng, and VILIP-1, are consistently found at elevated levels in the cerebrospinal fluid (CSF) of adults diagnosed with AD. Specifically, research by Maalmi et al. has demonstrated a correlation between higher serum NFL concentrations and the presence of diabetic sensorimotor polyneuropathy (DSPN) as well as nerve dysfunction [54, 55]. Likewise, VILIP-1, which is expressed in the pancreas, plays a role in modulating insulin secretion and the expression of the insulin gene [56]. Additionally, there was a notable reduction in Ng levels in hippocampal tissue compared to both the diabetic group and those subjected to endurance or endurance combined with supplementation.

Considering the role of the calmodulin-calcium signaling pathway in synaptic plasticity, several elements are being studied within the dentate gyrus of the hippocampus for their potential to influence cognitive functions [57]. Ng is highly expressed in cortical and hippocampal neurons, and its overexpression in CA1 hippocampal neurons has been shown to boost synaptic strength [58]. Unlike Ca^2+^/CaM-dependent enzymes, the binding affinity between CaM and Ng decreases in stimulated neurons as intracellular calcium concentrations rise. Moreover, Ng acts as a potent antioxidant protein, but it is vulnerable to oxidation when exposed to nitric oxide donors or following NMDA receptor activation [59]. Studies have shown that the absence of the Ng gene leads to cognitive impairments and long-term memory deficits in the hippocampus. Furthermore, Ng is transported from synaptic regions into the CSF, a process considered an early marker in AD progression. This also highlights Ng's potential as a biomarker for early diagnosis [60].

Systemic insulin resistance and diabetes have been linked to insulin resistance in the brain, which hinders nerve cell regeneration. Furthermore, insulin resistance is associated with increased levels of beta-amyloid in regions such as the frontal cortex and hippocampus [61]. On the other hand, the activation of insulin-degrading enzyme (IDE) facilitates the breakdown of amyloid β under normal physiological conditions. As a result, elevated amyloid β levels are closely tied to declines in memory and cognitive function among older adults, a crucial factor in aging [62].

Nerve injury is frequently accompanied by various pathological changes in brain tissue, suggesting possible interactions between abnormal protein deposits that contribute to cognitive decline and other clinical symptoms, potentially influenced by additional factors [63]. Growing evidence indicates that memory impairment is often initiated by subtle synaptic changes in the hippocampus that precede neurodegeneration. A reduction in synaptic density is linked to numerous neurological conditions, particularly AD [64].

Studies suggest that extended periods of exercise (spanning weeks or months) are associated with lower levels of neurodegenerative biomarkers during periods of rest [65]. High-intensity interval exercise has gained attention as a promising exercise model for investigating the effects of physical activity on neural biomarkers, especially in athletes [66]. Moreover, exercise has been shown to enhance neurogenesis and improve cognitive performance in the adult hippocampus [67].

Research indicates that adult neurogenesis in the hippocampus predominantly occurs near small blood vessels. Engaging in exercise promotes both vascular growth and cellular proliferation and repair within the dentate gyrus of the hippocampus [68]. As a result, endurance training enhances the functional reorganization of the brain's motor circuits, which involves dynamic processes of synaptic and neural reconstruction across the cerebellum, striatum, cerebral cortex, and hippocampus [69]. Lin et al. have noted that the depletion of Ng or bilateral common carotid artery stenosis adversely affects spatial memory, an effect that can be significantly alleviated through regular swimming exercise [4]. Their research also demonstrated that a swimming regimen restores levels of calmodulin and CaMKII, both vital for calcium signaling pathways that are otherwise disrupted by Ng depletion or carotid artery stenosis [4, 70]. Additionally, it has been shown that swim training influences inflammation and damage in white matter resulting from Ng knockout or carotid artery stenosis, consistent with our own findings. Several studies have reported reductions in NFL, Ng, and VILIP-1 levels associated with exercise, which closely aligns with our observations [25, 26].

In our current study, we found that resistance exercises performed at an intensity of 60% of MVCC led to a significant decrease in markers of molecular damage, potentially mitigating neuronal dysfunction in the brain. Furthermore, supplementation with UA resulted in a reduction of synaptic dysfunction markers. Notably, the combined approach of UA supplementation and exercise showed a synergistic effect on exploratory-locomotor behavior in rats. Thus, our research introduces a novel neuroprotective agent, illuminating how exercise contributes to brain health, functionality, and the treatment of specific neurodegenerative disorders. These results provide important insights into the complex mechanisms through which exercise and supplementation positively influence brain function. They point to the potential involvement of new proteins and pathways that facilitate memory, learning, synaptic plasticity, and resilience against neurological damage.

## 5. Limitations

This study focused on hippocampal biomarkers and general exploratory behavior using the OFT. While OFT efficiently assessed locomotor activity, spatial memory, and anxiety-like responses, cognition was not directly evaluated due to practical constraints (e.g., extended protocol duration required for Morris water maze in multigroup aging models). Future work will integrate dedicated cognition testing to complement biomarker findings. Nevertheless, our hippocampal biomarker findings provide novel insights into the neuroprotective mechanisms of exercise and UA against diabetes-driven neurodegenerative processes [37, 71].

## 6. Conclusion

Diabetes mellitus leads to neuronal damage in the hippocampus and contributes to neuronal and behavioral impairments by increasing levels of Ng, NFL, and VILIP-1, which are indicators of molecular damage. However, supplementation with UA along with participation in exercise especially resistance training can alleviate the harmful impacts of diabetes on hippocampal neuronal health and associated behaviors.

## Figures and Tables

**Table 1:** Insulin and serum glucose following 8 week exercise training and UA supplementation.

Variable	Group							*p* value	*F*
	C	DM	UA	ET	RT	ET + UA	RT + UA		
Glucose (mg/dL)	126.33 ± 28.72	385.5 ± 184.55	266.75 ± 97.61	343.66 ± 140.01	263.00 ± 160.26	354.25 ± 119.19	405.25 ± 43.29	0.115	2.126
Insulin (ng/mL)	0.13 ± 0.09	2.08 ± 0.98	0.29 ± 0.3	0.6 ± 0.68	0.09 ± 0.08	1.26 ± 0.55	0.61 ± 0.79	0.011^∗^	4.360

*Note:* Values are expressed as mean ± SEM. Data were analyzed by one-way ANOVA, *n* = 5 per group.

^∗^a statistically significant reduction in insulin expression (*p* < 0.01) in the RT and UA groups compared to the DM group.

## Data Availability

The data used to support the findings of this study are available from the corresponding author upon request.

## Nomenclature

STZ: Streptozotocin
HFD: High-fat diet
vVO_2max_: Velocity at maximal oxygen uptake
MVCC: Maximum voluntary carrying capacity
UA: Ursolic acid
NFL: Neurofilament light chain
Ng: Neurogranin
VILIP-1: Visinin-like protein 1
OFT: Open field test
BBB: Blood–brain barrier
T2DM: Type 2 diabetes mellitus
